# Supplementary material for: The functional landscape of the human ubiquitinome
Source: bioRxiv. 2025 Oct 8:2025.10.08.681129. Preprint. [Version 1] doi: 10.1101/2025.10.08.681129 (PMC12632403; doi:10.1101/2025.10.08.681129)
Supplement: Supplement 12 [file NIHPP2025.10.08.681129v1-supplement-12.pdf]

bioRxiv preprint doi: https://doi.org/10.1101/2025.10.08.681129; this version posted October 8, 2025. The copyright holder for this preprint (which was not certified by peer review) is the author/funder, who has granted bioRxiv a license to display the preprint in perpetuity. It is made available under aCC-BY 4.0 International license.

Supplementary figures

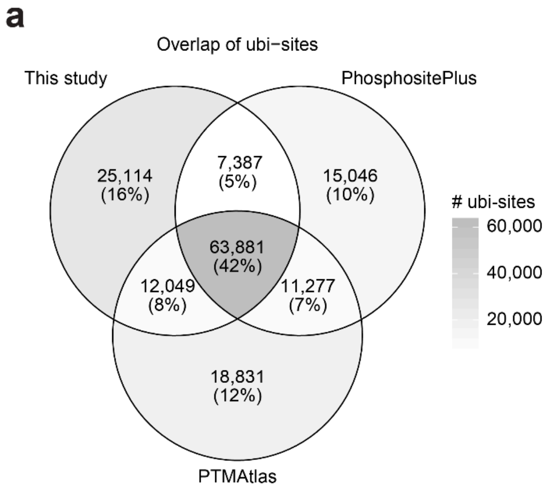

Figure S1: Additional analysis of reference ubiquitinome

A) The overlap of unique ubi-sites identified in our reference ubiquitinome, PhosphositePlus[17], and PTMAtlas[96] is shown.

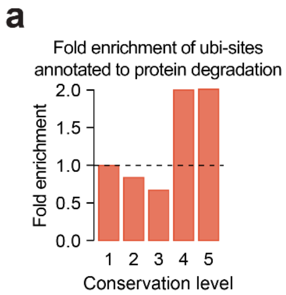

Figure S2: Additional analysis of conserved ubi-sites

A) The enrichment of ubiquitin sites annotated to perform degradation regulatory functions at each conservation level (PhosphositePlus[17]).

bioRxiv preprint doi: https://doi.org/10.1101/2025.10.08.681129; this version posted October 8, 2025. The copyright holder for this preprint (which was not certified by peer review) is the author/funder, who has granted bioRxiv a license to display the preprint in perpetuity. It is made available under aCC-BY 4.0 International license.

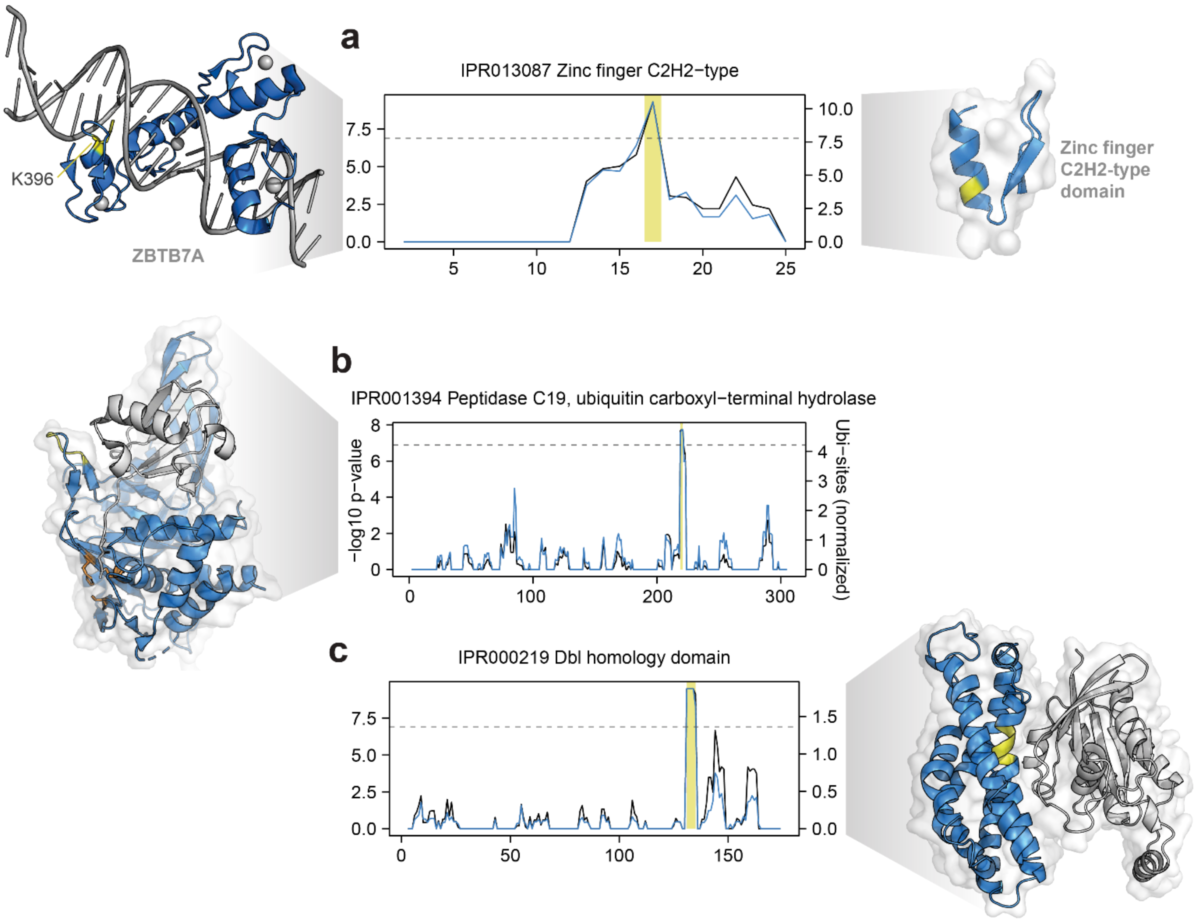

Figure S3: Additional ubi-site hotspots

A-C) Identification of ubi-site hotspots in three protein domains. The black line indicates the average number of ubi-sites observed across the domain sequence alignment within a rolling window, normalised by subtracting the number of ubi-sites expected by chance. The blue line indicates the p-value associated with the enrichment of ubi-sites at each alignment position. The horizontal line indicates a Bonferroni-corrected p-value cut-off of 0.01 (uncorrected p-value < 1.29×10^{-7}). Positions with a -log10 p-value above this cut-off and average number of phosphosites per window higher than 2 are classified as hotspot regions and highlighted with a yellow bar. Hotspot regions are mapped onto representative structures for each domain in yellow. In A) an example of a hotspot ubi-site located at the interface of a zinc finger with DNA is shown (K396 in ZBTB7A, PDB: 8E3E[97]). In B) the catalytic triad of the protease is coloured orange and a ubiquitin molecule covalently bound to the catalytic triad is coloured white. In C) the interaction partner Rac1 is coloured in white.

bioRxiv preprint doi: https://doi.org/10.1101/2025.10.08.681129; this version posted October 8, 2025. The copyright holder for this preprint (which was not certified by peer review) is the author/funder, who has granted bioRxiv a license to display the preprint in perpetuity. It is made available under aCC-BY 4.0 International license.

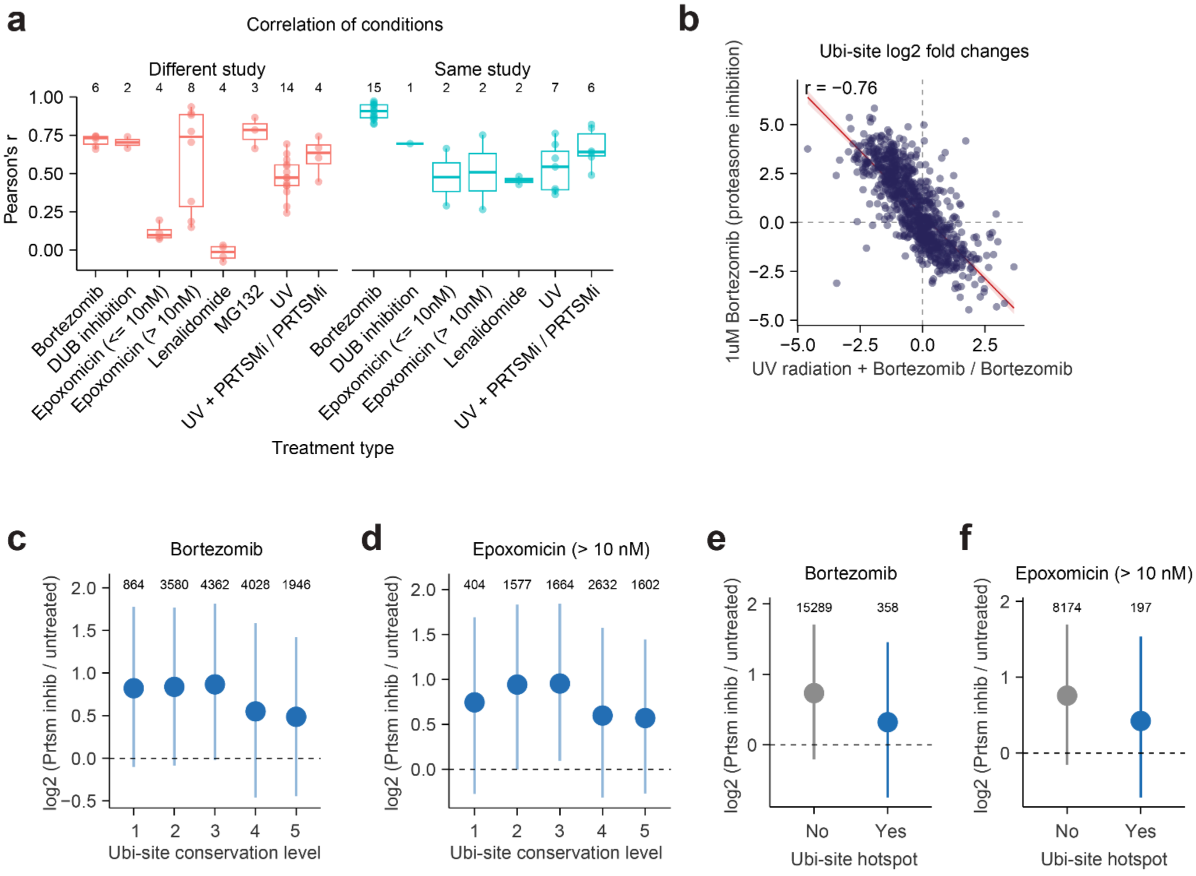

Figure S4: Additional analysis of quantitative ubiquitin proteomics data

A) Pearson's correlation was performed using ubi-site fold changes between pairs of conditions representing the same treatment. Pairs were separated based on whether the conditions came from the same study or different studies. The number of pairs in each group is indicated above boxplots. B) The correlation in ubi-site fold changes between Bortezomib treatment and UV radiation on a background of Bortezomib treatment. C-D) Mean log2-fold changes (± s.d.) of ubi-sites under proteasome inhibition (bortezomib or epoxomicin treatment), grouped by evolutionary conservation level. Final values were obtained by normalising and averaging log2 fold-changes from multiple experiments (see Methods). The number of ubi-sites in each group is indicated. Only epoxomicin treatments at > 10 nM were used since lower doses do not cause proteome-wide inhibition of protein degradation[55]. E-F) As in C-D), for sites in ubi-site domain hotspots.

bioRxiv preprint doi: https://doi.org/10.1101/2025.10.08.681129; this version posted October 8, 2025. The copyright holder for this preprint (which was not certified by peer review) is the author/funder, who has granted bioRxiv a license to display the preprint in perpetuity. It is made available under aCC-BY 4.0 International license.

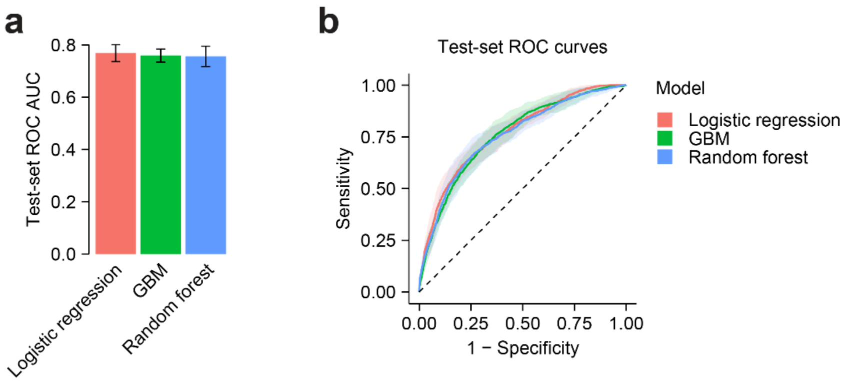

Figure S5: Training a machine-learning based ubiquitin site functional score
A-B) Three classifier architectures were trained to separate unannotated ubi-sites from ubi-sites annotated to non-degradation roles. Classifiers were trained and evaluated through a five-fold train-test split, with hyperparameter optimization performed during each training iteration via five-fold cross-validation. A) shows ROC curve AUCs on each test set (error bars indicate standard deviation) while B) shows averaged ROC curves (shading indicates standard deviation).

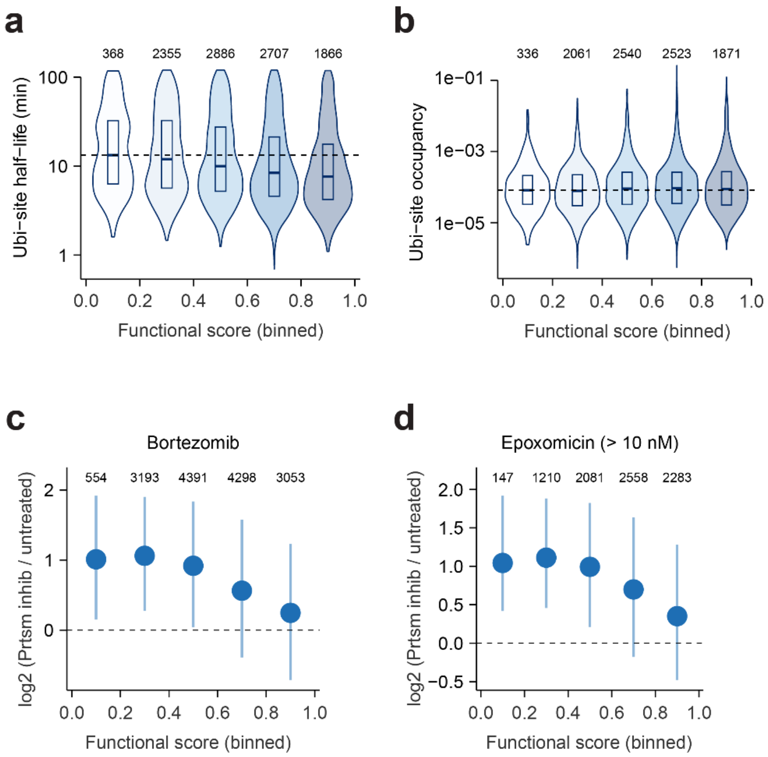

Figure S6: Additional characterisation of the ubi-site functional score
A-B) Measurements of A) ubi-site half-life and B) ubi-site occupancy in *Prus et al.*[16], binned into functional score ranges. Numbers of quantified ubi-sites per bin are shown. The median of the lowest functional score bin is shown as a dotted line. C-D) Mean log2-fold changes (± s.d.) of ubi-sites under

bioRxiv preprint doi: https://doi.org/10.1101/2025.10.08.681129; this version posted October 8, 2025. The copyright holder for this preprint (which was not certified by peer review) is the author/funder, who has granted bioRxiv a license to display the preprint in perpetuity. It is made available under aCC-BY 4.0 International license.

proteasome inhibition (bortezomib or epoxomicin treatment), binned into functional score ranges. Numbers of quantified ubi-sites per bin are shown. Final values were obtained by normalising and averaging log2 fold-changes from multiple experiments (see Methods). Only epoxomicin treatments at > 10 nM were used since lower doses do not cause proteome-wide inhibition of protein degradation[55].

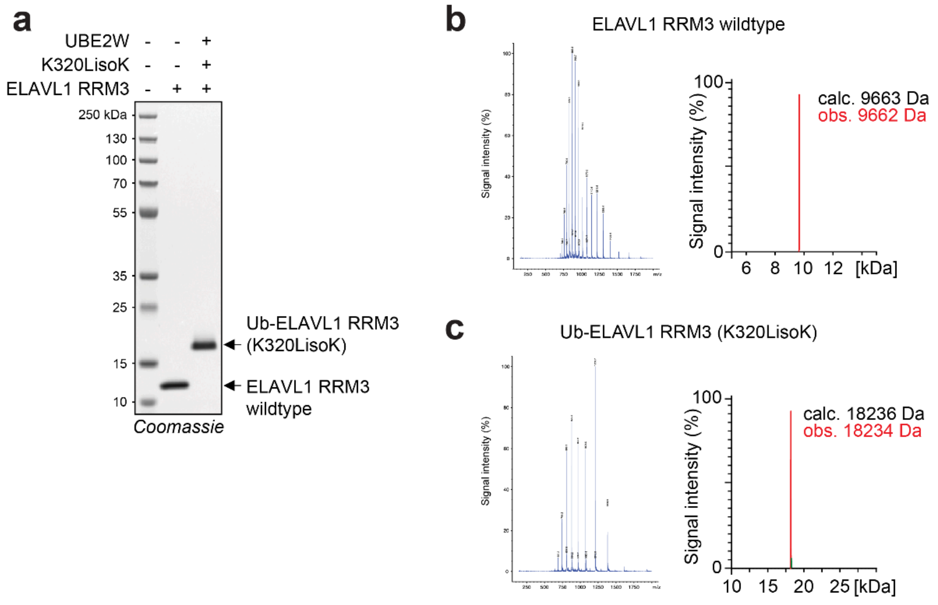

Figure S7: Confirmation of ELAVL1 RRM3 constructs
A) Coomassie-stained SDS-PAGE of purified ELAVL1 constructs: Wildtype ELAVL1 RRM3 and ubiquitinated ELAVL1 RRM3 (K320LisoK). The cropped blot is shown in Fig. 5e. B-C) LC-MS measurement of purified B) ELAV1 RRM3 and C) Ub-ELAVL1 RRM3. Left: non-deconvoluted spectra, right: deconvoluted spectra.

Supplementary tables and data

Table S1: Datasets in the human reference ubiquitinome

Table S2: The human reference ubiquitinome

Table S3: Pan-species ubiquitin proteomics datasets
All_Experiments_nonHs: Meta-data from proteomics experiments in non-human species. Quantitative_Conditions: Meta-data of human quantitative proteomics data.

Table S4: Pan-species ubiquitin proteomics sites

Table S5: Ubiquitin site domain hotspots

bioRxiv preprint doi: https://doi.org/10.1101/2025.10.08.681129; this version posted October 8, 2025. The copyright holder for this preprint (which was not certified by peer review) is the author/funder, who has granted bioRxiv a license to display the preprint in perpetuity. It is made available under aCC-BY 4.0 International license.

hotspotSummary: Summary of hotspots. hotspotSites: All ubi-sites found in hotspots. hotspotRegulatorySites: Hotspot ubi-sites with regulatory annotations in PhosphositePlus.

Table S6: Functional scores and features used for training

Table S7: Ubiquitin sites annotated to degradative and non-degradative functions in PhosphositePlus

Table S8: Ubiquitin sites in nuclear localisation signals or associated with protein activity

Table S9: Yeast chemical genetics experimental data
conditionDescriptions: Descriptions of conditions used in growth screens. MutantDescriptions: Yeast strains used in growth screens. Sscores: S-scores and Q-values from chemical genetics experiments. primers: Primer sequences used for CRISPR-Cas9 generation of lysine mutants. donors: Donor sequences used for CRISPR-Cas9 generation of lysine mutants.

Table S10: Plasmids used in this study

Data S1: Ubi-site hotspots
Visualisation of all ubi-site hotspots with a hotspot found in a representative crystal structure.
